# Transoral Excision of an Intracordal Schwannoma: A Case Report and Review

**DOI:** 10.7759/cureus.57823

**Published:** 2024-04-08

**Authors:** Justin B Fong, Christopher G Tang

**Affiliations:** 1 Otolaryngology - Head and Neck Surgery, Geisel School of Medicine at Dartmouth, Lebanon, USA; 2 Otolaryngology - Head and Neck Surgery, Kaiser Permanente San Francisco, San Francisco, USA

**Keywords:** otolaryngology, vocal folds, transoral excision, laryngology, schwannoma

## Abstract

We discuss a novel approach to resecting a large 1.5 cm intracordal schwannoma via direct laryngoscopy with combined endoscopic and microlaryngoscopic techniques. Removing relatively bulky masses within the vocal cord soft tissue can be challenging secondary to difficult visualization of the operative field during direct laryngoscopy. We describe a case where a bulky atypical spindle cell schwannoma was removed via direct laryngoscopy via combined endoscopic and microlaryngoscopic techniques. The tumor obstructed 40% of the visual field of the laryngoscope. In this case, a 44-year-old female presented to the head and neck surgery clinic with 1.5 years of progressive hoarseness. On fiberoptic laryngoscopy, a mass was noted medializing the right true vocal cord. The patient was taken to surgery and after intubation and suspension with a Dedo laryngoscope, the mass was removed trans-orally through the laryngoscope with visualization using a combination of rigid and flexible endoscopy as well as with a microscope. Although visualization can sometimes be reduced using direct laryngoscopy, surgical excision of relatively large laryngeal masses can be performed in selected cases without the need to approach the masses trans-cervically.

## Introduction

Schwannomas or neurilemmomas are neurogenic tumors that originate from the Schwann cells that make up the myelin sheaths in the peripheral nervous system. Schwannomas are benign and encapsulated tumors that can present anywhere in the peripheral nervous system, with 25%-45% occurring within the head and neck region [1]. These tumors are characterized by slow growth and immunoreactivity to S-100, a protein commonly used to identify a Schwann cell, melanocyte, or chondrocyte origin of tumors [1]. Laryngeal manifestations of schwannoma are uncommon, and there are only about 75 reported cases in English language literature. The majority of reported laryngeal schwannomas arise from the aryepiglottic folds or the arytenoids. The majority of these patients present with dysphonia and are preferentially managed through surgical intervention [1,2]. As a rare manifestation of an uncommon disease, very few reported cases of intracordal schwannoma are in the literature. We present a case of intracordal schwannoma and a comprehensive review of the literature.

The authors have attained written informed consent from the patient for the below case and associated images to be published.

## Case presentation

Initial presentation 

A 43-year-old otherwise healthy female was referred to the head and neck surgery clinic for progressive hoarseness over the past 1.5 years. The patient had no symptoms other than a moderate vocal fry. Fiberoptic transnasal laryngoscopy revealed mobile vocal cords with a right intracordal mass. The patient was then referred to a laryngologist for surgical excision.

Treatment pathway

**Figure 1:**
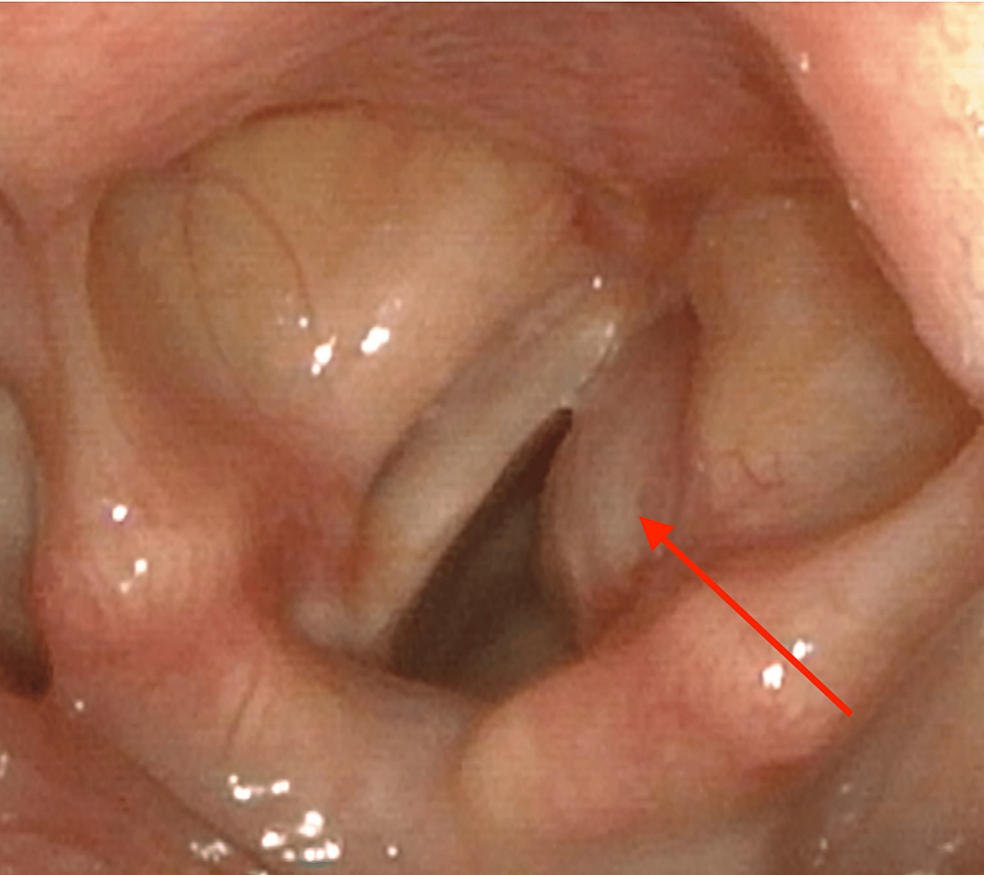
Preoperative laryngoscopy view of the right vocal cord with what appears to be an intracordal mass causing medialization.

**Figure 2:**
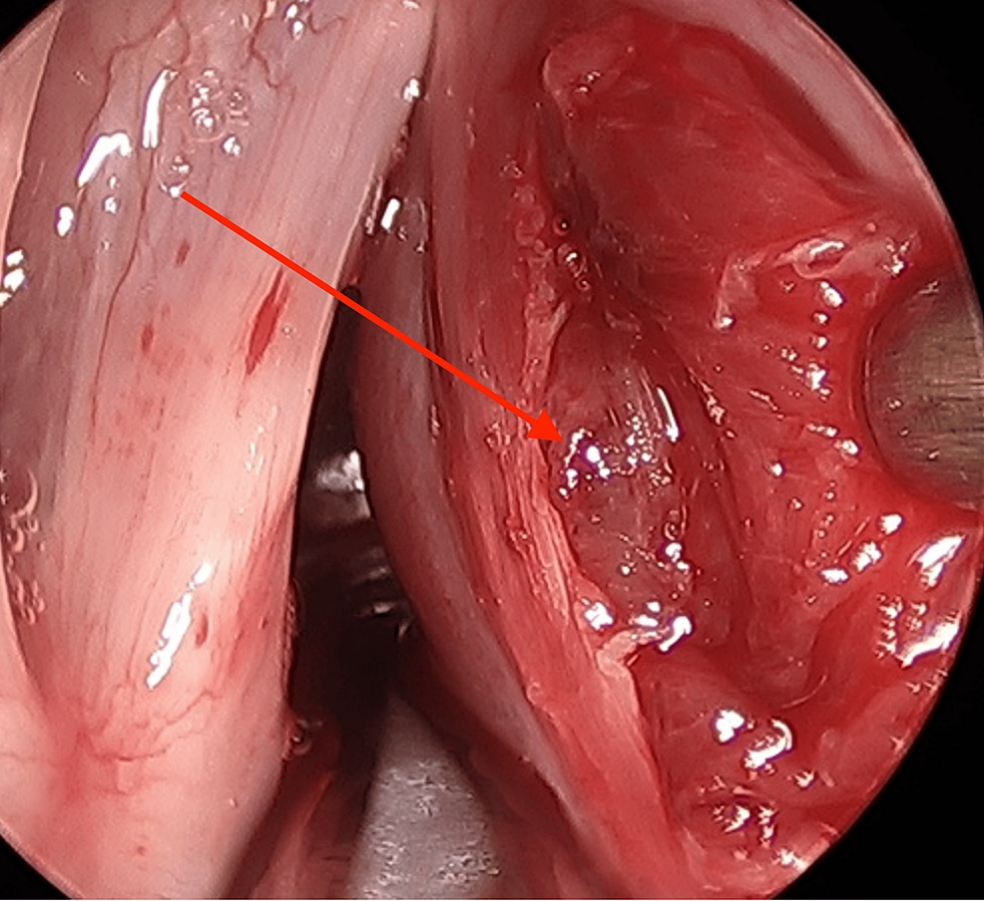
Intraoperative view of the mass being exposed within the right vocal cord.

Preoperative stroboscopy was performed which revealed the mass and a poor mucosal wave with decreased amplitude, out-of-phase waves when periodic, although most of the time the cords were aperiodic given the presence of the mass. The patient was scheduled for a direct microlaryngoscopy and excisional biopsy. On direct visualization of the cords, the anterior two-thirds of the right vocal cord was found to be protuberant and medialized (Figure 1). Using a sickle knife, an incision was made extending anterior to posterior, lateral to the vocal ligament on the superior surface of the right vocal cord. Retraction of the medial mucosa and vocal cord muscle using triangle forceps revealed the underlying mass. Using the microflap technique and blunt instrumentation (spatula), the mass was freed from the surrounding tissues and removed (Figure 2).

Intraoperative consultation with pathology suggested a spindle cell tumor on frozen sectioning. The incision was irrigated, and hemostasis was confirmed. The wound was reapproximated, and the right thyroarytenoid muscle was left intact. The patient woke from anesthesia without complications. The final pathology of the excised mass was consistent with schwannoma with positive S-100 immunoreactivity. 

Outcomes

**Figure 3:**
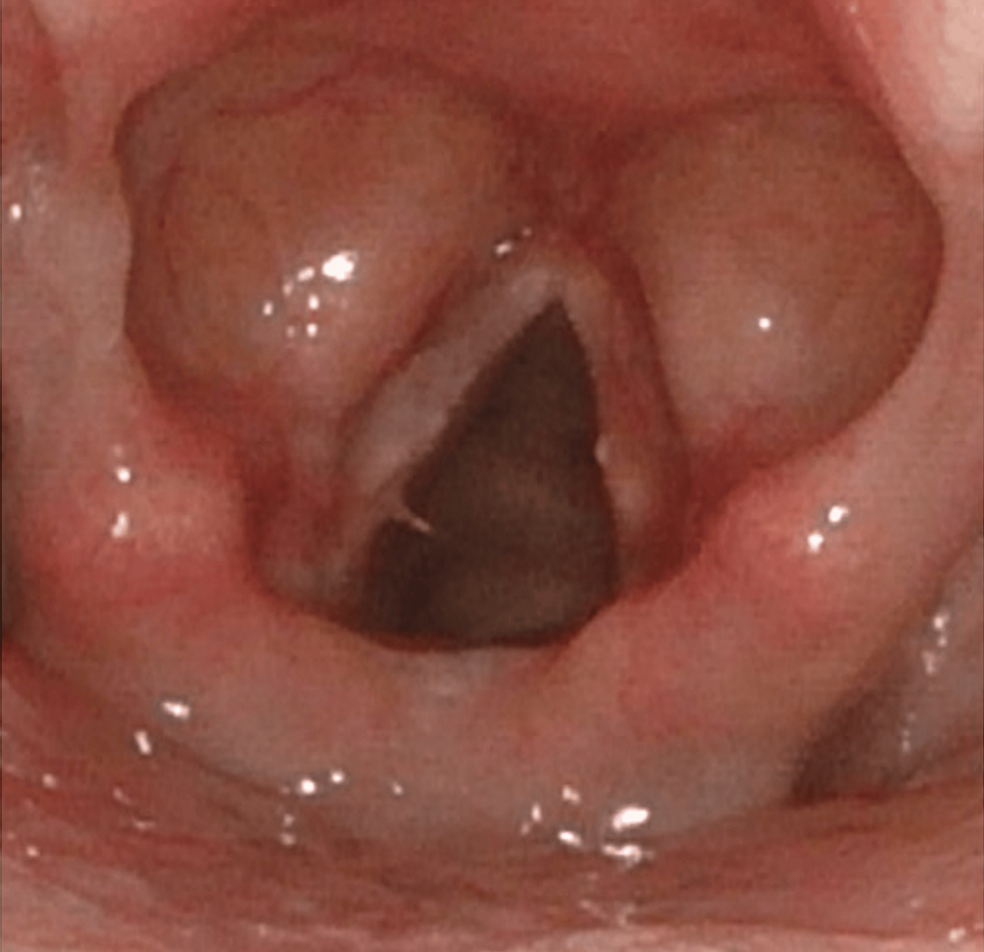
Two-week postoperative laryngoscopy revealed well-healed vocal cords with no evidence of mass in the right vocal cord.

The immediate postoperative course was uneventful. Two weeks after surgery, her vocal cords were mobile, and she had an improved phonation (Figure 3). At eight months, the patient followed up with excellent bilateral vocal cord motility, good glottic closure, excellent mucosal wave with normal amplitude, periodic, in-phase vibrations, and normal voice.

## Discussion

Literature review

A comprehensive search of the Medline, Scopus, and Web of Science databases was conducted in January 2024. The goal was to identify all documented cases of intracordal schwannoma in English literature from January 1990 through December 2023. Titles and abstracts were searched for the following: vocal cord, intracordal, vocal fold, neurilemmoma, schwannoma, nerve sheath tumor, and Schwann cell tumor. MeSH terms included were ("Neurilemmoma"[Mesh) and ("Vocal Cords"[Mesh]). The search of databases revealed 223 articles after duplications were removed. Titles and abstracts were reviewed for relevant articles. Thirteen full-text articles were reviewed, of which two articles contained previously reported cases. A total of 11 articles were included.

A total of 11 cases of intracordal schwannoma were identified in 11 articles (Table 1). We added one case to this report. The mean age on presentation was 36 years old, with no observed preponderance between males and females. All but one patient presented with a chief complaint of dysphonia or hoarseness (11, 92%). The next most common presenting symptom was dyspnea on exertion (two cases) followed by foreign body sensation (one case). Tumor size was reported in six of 12 cases and ranged from 7 to 20 mm. Due to heterogeneous data, we are unable to make associations between the size of the tumor and presenting symptoms. Six patients presented with a right vocal cord tumor, while five patients presented with a left vocal cord tumor (Table 1).

**Table 1:** English language literature review of all reported intracordal schwannomas with presentation, location, treatment, size, and outcome. NR, not recorded; FOB, foreign object

Case	Age	Sex	Presentation	Location	Treatment	Mass size (widest)	Stain	Outcome	Follow-up	Reference	Date
1	44	F	Dysphonia	Vocal cord, unspecified	Transoral excision and microlaryngoscopy	NR	S-100	NR	NR	Dekker and Haidar [3]	1994
2	32	F	Dysphonia, six months	Vocal cord, left posterior	Transoral excision, endoscopic, and laser	7 mm	S-100	Healthy	48 months	Zbären and Markwalder [4]	1999
3	27	M	Dysphonia, two years	Vocal cord, left	Transoral excision, microlaryngoscopy	19 mm	S-100	Healthy	15 months	Tzagkaroulakis et al. [5]	2003
4	26	F	Dysphonia, seven years	Vocal cord, right	Transoral excision, microlaryngoscopy	NR	S-100	Healthy	Four months	Taylor et al. [6]	2006
5	19	M	Dysphonia, one year; FOB sensation	Vocal cord, right	Surgical excision; external	NR	S-100	Healthy	NR	Chandrashekhara et al. [7]	2010
6	37	M	Dyspnea on exertion	Vocal cord and ventricular fold, left	Surgical excision; external	NR	S-100	NR	NR	Ueha et al. [8]	2011
7	30	M	Dysphonia	Vocal fold, right anterior	Transoral excision, microlaryngoscopy	20 mm	S-100	Healthy	Six months	Kharytaniuk [9]	2014
8	60	F	Dysphonia	Vocal cord and ventricular fold, left	Transoral excision, microlaryngoscopy	15 mm	NR	Unknown	None	Romak et al. [10]	2017
9	38	M	Dysphonia, dyspnea on exertion	Vocal cord, left anterior	Transoral excision, endoscopic, laser	20 mm	S-100	Healthy	Eight months	Singh et al. [11]	2018
10	13	F	Dysphonia, six months	Vocal cord, right	Transoral excision, microlaryngoscopy	NR	S-100	Healthy	Three months	Swain et al. [12]	2020
11	60	M	Dysphonia	Vocal cord, right	Transoral excision, endoscopic, and laser	NR	NR	Healthy	Eight months	Tritter and Sadoughi [13]	2021
12	43	F	Dysphonia, one year	Vocal cord, right	Transoral excision, microlaryngoscopy	15 mm	S-100	Healthy	Eight months	Fong	2022

All included cases were treated with surgical excision of the tumor. The majority of cases were treated with transoral excision via direct microlaryngoscopy surgery (10, 83%). Of those treated with microlaryngoscopy surgery, three patients (30%) were treated via CO_2_ laser endoscopy. Due to bulky tumors, two patients were treated by external approach surgical resection. There was no observed difference in outcomes for each surgical approach; however, our analysis is limited due to variations in reporting and follow-up standards across individual case reports. Follow-up data were available for nine cases, all of which reported full recovery of voice and no evidence of disease.

Case discussion

Schwannomas are uncommon benign tumors that arise from the Schwann cells of the peripheral nervous system. Intracordal laryngeal schwannomas are a very rare subtype of schwannomas with only 12 reported cases in English language literature since 1990. This case report and review has assembled the largest English language review of the clinical presentation and treatment of intracordal schwannoma.

With such a small number of cases, making definitive epidemiological predictions is not possible. However, the seeming lack of sex predilection and strong age preference aligns with prior meta-analyses done of all laryngeal schwannoma [1,2].

Like many other vocal cord diseases, intracordal schwannoma most commonly first presents with dysphonia and impaired vocal cord mobility. The associated mass on the vocal cord is easily visualizable by in-office flexible laryngoscopy. Differential diagnosis should include both benign and malignant processes including vocal cord polyp, vocal cord cyst, and submucosal carcinoma. The final diagnosis of intracordal schwannoma is normally made after excision through pathological analysis and immunoreactivity to S-100 protein.

Like other laryngeal schwannomas, the preferred choice of therapy for intracordal schwannoma is complete surgical resection. Three main approaches to the excision of intracordal schwannomas have been described: microlaryngoscopy excision with a CO_2_ laser, microlaryngoscopy excision without a CO_2_ laser, and external approach surgical excision. Patients undergoing any of these surgeries for intracordal schwannomas have a very favorable prognosis, as there have been no reported deaths, and all patients followed have exhibited normal phonation with no evidence of disease.

## Conclusions

Intracordal schwannoma is a very rare tumor, primarily treated by surgical removal. With the addition of our patient case, this current study is the largest review of the clinical presentation and treatment of intracordal schwannoma. Transoral resection is feasible in the majority of cases with no reported recurrences. This case describes a multimodal approach to remove the tumor transorally, sparing the patient of an external approach.
